# 

**Published:** 1896-05-16

**Authors:** 


### EARLY EDITION.

**Notices of Births, Deaths, and Marriages** are inserted in this column at a charge of 2s. 6d. for an announcement not exceeding four lines.

**Notice to Advertisers and Subscribers.**
All Advertisements, Orders for Copies of The Hospital and Business Communications should be addressed to **THE MANAGER, (Not to The Editor),** The Hospital, 428, Strand, London, W.C.

The Cost of Subscription, post free, is as follows:—Per week, 2½d.; per quarter, 2s. 8d.; per half-year, 5s. 3d.; per annum, 10s. 6d. Foreign:—Per Annum. 15s. 2d.; payable, in all cases, in advance.

Ready Shortly.
**"Burdett's Hospitals and Charities, 1896."**
Seventh year of publication. Over 1,000 pp. Scarlet cloth, gilt lettered, **Price 5s.** A most complete guide to British, Colonial, Indian, and American Hospitals, Dispensaries, Nursing and Convalescent Institutions, and Asylums. A few complete sets of the preceding six issues of the "Annual" may still be obtained on application to the Publishers, The Scientific Press (Limited), 428, Strand, London, W.C.

**NOTICE.—Vol. XIX. of "The Hospital" (October, 1895, to March, 1896), in handsome cloth binding, is now ready and on sale at the Office of this Journal. Price 7s. 6d. Cases for binding the Numbers contained in this Volume, 1s. 6d. Index to Vol. XIX., 2½d., post free.**

**The Monthly Part for May is now ready, price 6d., by post, 8d.**

### The Student of Medicine.

### APPOINTMENTS.

J. R. Benson, M.R.C.S.Eng., L.R.C.P.Lond., appointed House Surgeon to King's College Hospital. C. S. Bond, B.A.Camb., M.B., B.C., appointed Assistant Medical Officer to the Chelsea Infirmary. H. B. Burridge, M.R.C.S.Eng., F.R.C.P.Lond., appointed House Surgeon to King's College Hospital. A. F. G. Codd, M.B.Durh., F.R.C.S.Eng., reappointed Medical Officer of Health to the Bromley Urban District Council. Dr. Cook, appointed Consulting Physician to the Pembrokeshire County Infirmary. F. Dale, M.D.Cantab., F.R.C.S.Eng., appointed Honorary Ophthalmic and Aural Surgeon to the Scarborough Hospital and Dispensary. C. C. J. Erhardt, M.R.C.S.Eng., L.R.C.P.Lond., appointed House Surgeon to King's College Hospital. A. H. Evans, appointed Senior House Physician to Westminster Hospital. J. A. Ewan, M.D.Edin., appointed Medical Officer to the Kesteven and Grantham Asylum. T. C. Gray, F.R.C.S.Eng., L.R.C.P.Lond., appointed Surgeon to the Great Northern Railway Company. W. Griffith, M.B., C.M. Edin., appointed Consulting Physician to the Pembrokeshire County Infirmary. W. H. Hargreaves, M.R.C.S., L.R.C.P.,

### THE STUDENT OF MEDICINE—*continued.*

appointed Obstetric House Physician to Middlesex Hospital. D. Havard, M.D.St.And., L.R.C.P., M.R.C.S., appointed Consulting Physician to the Pembrokeshire County Infirmary. F. H. Jacob, M.R.C.S Eng., L.R.C.P.Lond., appointed Assistant House Physician to King's College Hospital. G. J. Jenkins, M.B., C.M., appointed Medical Officer to the Craiglockhart Poorhouse of the Edinburgh Parish Council. S. A. Jolly, L.R.C.P., L.R.C.S.Edin., appointed Medical Officer for the Acton District of the Brentford Union. G. H. Lansdown, M.R.C.S.Eng., L.R.C.P. Lond., appointed House Physician to King's College Hospital. Dr. McHale, appointed Medical Officer for the Laharvane District of the Castlebar Union. T. A. Papillon, F.R.C.S.Edin., appointed Ophthalmic Surgeon to the Hastings, St. Leonards, and East Sussex Hospital. E. Playfair, M.B.Lond, M.R.C.S. Eng., L.R.C.P.Lond., appointed House Accoucheur to King's College Hospital. H. P. J. Price, M.R.C.S.Eng., L.S.A., appointed Consulting Physician to the Pembrokeshire County Infirmary. D. A. Reid, M.D.Edin., M.R.C.S., appointed Consulting Physician to the Pembrokeshire County Infirmary. E. A. Saunders, M.R.C.S.Eng., L.S.A., appointed Consulting Physician to the Pembrokeshire County Infirmary. W. R. Scott, M.D., M.Ch.R.U.I., L.A.H.Dubl., appointed Medical Officer of Health for Bloemfontein Orange Free State. H. Sinigar, M.B. Lond., M.R.C.S., L.R.C.P., appointed Assistant Medical Officer to the Birmingham Workhouse Infirmary. A. Stanley, M.D. Lond., M.R.C.S., F.R.C.P., late Resident Medical Officer, Sussex County Hospital, appointed Assistant Medical Officer at the North-Western Hospital of the Metropolitan Asylums Board. W. H. Symons, D.P.H., F.I.C., appointed Medical Officer of Health to the Bath Urban Sanitary Authority. R. S. Thomas, M.A., M.B., B.C.Cantab., M.R.C.S.Eng., L.R.C.P.Lond., appointed Medical Officer to the Exmouth Dispensary. P. C. E. Tribe, M.R.C.S.Eng., L.R.C.P.Lond., appointed Assistant Accoucheur to King's College Hospital. Dr. Williams, appointed Consulting Physician to the Pembrokshire County Infirmary.

### VACANCIES.

#### Resident Medical Officers.

Bucks County Lunatic Asylum, Stone, near Aylesbury. Assistant Medical Officer; unmarried. Salary £100 per annum, with board and furnished apartments in the asylum. Applications to Mr. Wm. Crouch, Clerk to the Visiting Committee, County Hall, Aylesbury, by May 16th.

Bristol Eye Hospital.—House Surgeon. Salary £60 per annum, with board and residence. Applications to Mr. T. Hampton, Secretary, by May 19th.

Chichester Infirmary.—House Surgeon. Salary £80 per annum, with board, lodging, and washing. Applications to the Secretary by May 18th.

County Asylum, Dorchester.—Senior Assistant Medical Officer. Age not under 26, and unmarried. Salary £150, rising to £200 per annum. Applications to the Medical Superintendent before May 20th.

St. Andrew's Hospital for Mental Diseases, Northampton.—Junior Assistant Medical Officer; unmarried; doubly qualified. Salary £150 per annum, with board, furnished apartments, and washing. Applications to the Medical Superintendent by May 18th.

Victoria Hospital for Sick Children, Queen's Road, Chelsea, S.W.—House Physician for In-patients. Honorarium £50 per annum, with board and lodging. Appointment for twelve months. Assistant Physician to Out-patients. Appointment for five years, but eligible for re-election. Applications to the Secretary by May 16th.

#### Non-Resident Medical Officers.

County Borough of Gateshead.—Medical Officer of Health. Salary £300 per annum, rising £25 yearly to £350. Candidate elected will also be appointed Medical Officer of the Corporation Hospital for Infectious Diseases, for which an additional salary of £50 per annum will be paid. Must be doubly qualified, and must reside in the Borough. Appointment for twelve months, but eligible for re-election. Applications to W. Swinburne, Town Clerk, Town Hall, Gateshead, by May 16th.

London Throat Hospital, 204, Great Portland Street, W.—Non-resident House Surgeon. Appointment for six months. Salary at the rate of 25 guineas per annum. Applications to the Honorary Secretary of the Medical Committee by May 20th.

